# LCZ696 Ameliorates Oxidative Stress and Pressure Overload-Induced Pathological Cardiac Remodeling by Regulating the Sirt3/MnSOD Pathway

**DOI:** 10.1155/2020/9815039

**Published:** 2020-09-17

**Authors:** Shi Peng, Xiao-feng Lu, Yi-ding Qi, Jing Li, Juan Xu, Tian-you Yuan, Xiao-yu Wu, Yu Ding, Wen-hua Li, Gen-qing Zhou, Yong Wei, Jun Li, Song-wen Chen, Shao-wen Liu

**Affiliations:** ^1^Department of Cardiology, Shanghai General Hospital, School of Medicine, Shanghai Jiao Tong University, Shanghai, China; ^2^Department of Cardiology, Shanghai Chest Hospital, Shanghai Jiao Tong University, China; ^3^Department of Ultrasound, The Second Affiliated Hospital of Soochow University, Suzhou, China; ^4^Department of Cardiology, Affiliated Wujin Hospital of Jiangsu University, China

## Abstract

**Aims:**

We aimed to investigate whether LCZ696 protects against pathological cardiac hypertrophy by regulating the Sirt3/MnSOD pathway.

**Methods:**

*In vivo*, we established a transverse aortic constriction animal model to establish pressure overload-induced heart failure. Subsequently, the mice were given LCZ696 by oral gavage for 4 weeks. After that, the mice underwent transthoracic echocardiography before they were sacrificed. *In vitro*, we introduced phenylephrine to prime neonatal rat cardiomyocytes and small-interfering RNA to knock down Sirt3 expression.

**Results:**

Pathological hypertrophic stimuli caused cardiac hypertrophy and fibrosis and reduced the expression levels of Sirt3 and MnSOD. LCZ696 alleviated the accumulation of oxidative reactive oxygen species (ROS) and cardiomyocyte apoptosis. Furthermore, Sirt3 deficiency abolished the protective effect of LCZ696 on cardiomyocyte hypertrophy, indicating that LCZ696 induced the upregulation of MnSOD and phosphorylation of AMPK through a Sirt3-dependent pathway.

**Conclusions:**

LCZ696 may mitigate myocardium oxidative stress and apoptosis in pressure overload-induced heart failure by regulating the Sirt3/MnSOD pathway.

## 1. Introduction

Cardiac hypertrophy is a pathological remodeling process of the heart characterized by hypertrophied cardiomyocytes, interstitial fibrosis, perivascular fibrosis, and decreased cardiac compliance. The process can eventually lead to malignant arrhythmias, heart failure, and even sudden cardiac death [1, 2]. Heart failure is the final stage of all the cardiovascular diseases and is currently a heavy burden for national medical and health services. Although several treatment options, such as CRT (Cardiac Resynchronization Therapy), exist, heart failure-associated morbidity and mortality are still on the rise. Thus, there is a need to identify novel therapeutic targets against the condition [3].

LCZ696 is an angiotensin-receptor-neprilysin inhibitor (ARNI) consisting of the neprilysin inhibitor sacubitril (AHU377) and angiotensin-receptor blocker (ARB) valsartan [4]. Recent clinical trials have revealed that LCZ696 is superior to enalapril in reducing mortality and rehospitalization rate in patients with chronic heart failure [5, 6]. Moreover, LCZ696 can reduce both sudden cardiac death and deaths from progressive heart failure compared to angiotensin-converting enzyme inhibitors (ACEI), such as enalapril [5]. Although LCZ696 is effective in the treatment of heart failure [7, 8], the specific mechanism underlying its role in long-term pressure overload-induced cardiac hypertrophy and sequential heart failure remains unknown.

The intracellular generation and scavenging of reactive oxygen species (ROS) are usually in a homeostasis state under normal physiological conditions. However, under various pathogenic stimuli, such as irradiation, anticancer agents, and aromatic hydrocarbons, the accumulation of ROS far exceeds the clearance capacity of cells. Consequently, various macromolecules suffer from oxidative stress damage, including lipid peroxidation, oxidative damage to DNA, protein oxidation, and monosaccharide oxidation [9]. Reactive oxygen species can enhance the progression of cardiac hypertrophy and heart failure [10–13]. Cardiac hypertrophy causes elevated mitochondrial ROS levels, which in turn induces myocardium apoptosis and further aggravates hypertrophy, ending up in a vicious circle [14]. ROS can activate multiple signaling cascades during cardiac hypertrophy. As such, impeding ROS and breaking the vicious cycle may hold promise for improving cardiac hypertrophy [15].

Sirtuins are a family of nicotinamide adenine nucleotide- (NAD^+^-) dependent class III histone deacetylases (HDACs) consisting of seven homologues. Among the homologues, sirtuin 3 (Sirt3) is the most extensively studied because of the decisive role it plays in a variety of diseases. Sirt3 is highly expressed in tissues requiring high energy metabolisms, especially in the heart tissues. The role of Sirt3 in mitochondrial dysfunction and redox homeostasis has been extensively demonstrated [16, 17]. Recent studies have shown that Sirt3 plays a crucial role in defending mitochondria from oxidative damage [18]. Also, it has been elaborated that Sirt3 deficiency exacerbates cardiac hypertrophy and heart failure in transverse aortic constriction mice, whereas Sirt3 overexpression protects against maladaptive ventricular remodeling [19, 20]. Given this, we hypothesized that the potential mechanisms underlying the beneficial effects of LCZ696 on pathological cardiac remodeling could be mediated by the Sirt3-dependent pathway.

The results of the present study showed that LCZ696 upregulated Sirt3 expression levels both in hypertrophied cardiac myocytes with transverse aortic constriction and cardiomyocytes induced with phenylephrine (PE). Also, we observed that the cardioprotective effects of LCZ696 were partly mediated by the Sirt3-dependent pathway.

## 2. Materials and Methods

### 2.1. Reagents

The following antibodies were purchased from Cell Signaling Technology: anti glyceraldehyde-3-phosphate dehydrogenase (GAPDH, anti-Sirt3 rabbit monoclonal antibody, anti-MnSOD rabbit monoclonal antibody, anti-COX IV rabbit monoclonal antibody, anti-AMPK rabbit monoclonal antibody, and anti phospho-AMPK rabbit monoclonal antibody. Mouse monoclonal anti sarcomeric alpha-actinin, rabbit monoclonal anti-Bcl-2 antibody, rabbit monoclonal anti-Bax antibody, mouse monoclonal anti-3-nitrotyrosine, and rabbit polyclonal anti-4 hydroxynonenal were purchased from Abcam (Cambridge, MA, United States). LCZ696 was purchased from MedChemExpress (Monmouth Junction, NJ, USA) and dissolved in corn oil.

### 2.2. Animals and Treatments

Eight-week-old male C57BL/6 mice and neonatal Sprague-Dawley (SD) rats were obtained from GemPharmatech Co. Ltd. (Nanjing, China). The animals had free access to food and drinking water. All the animal experiments were conducted as per the guidelines of the care and use of laboratory animals of the Shanghai Jiao Tong University School of Medicine.

Transverse aortic constriction surgery was performed as previously described [21]. In brief, mice were anaesthetized with a mixture of ketamine (100 mg/kg) and xylazine (5 mg/kg) through intraperitoneal injection. The lack of toe pinching reflex indicated successful anaesthesia. The chest cavity was opened to the second intercostal space, and the adipose and connective tissue around the aortic arch was gently separated. The constriction was applied between the left common carotid artery and the innominate artery, by tying a 6-0 nylon suture ligature against a 27-gauge needle. The thorax was closed using a 6-0 suture, and mice were left to rest on a warming pad until they were fully awake. The sham-operated mice underwent the same operation procedure devoid of aortic ligation. After the ligation, the mice were randomly divided into four groups: sham+vehicle group, sham+LCZ696 group, TAC+vehicle group, and TAC+LCZ696 group. To determine the role of LCZ696 in pathological cardiac remodeling, mice were administered with LCZ696 at a dose of 20 mg/kg/d through gavage. The dosage of LCZ696 was selected according to previous studies [22, 23]. The vehicle group was given the same volume of corn oil. After 4 weeks, hearts were snap-frozen in liquid nitrogen, then kept at -80°C for subsequent analyses.

### 2.3. Hemodynamic and Echocardiographic Measurements

The VEVO 2100 echocardiography system (Visual Sonics Inc., Toronto, Canada) was used to perform transthoracic echocardiography as per the methods described previously. Briefly, 4 weeks after TAC surgery, the mice were anaesthetized with 2.5% isoflurane. Subsequently, M-mode echocardiography was used to obtain left ventricular internal diameter at end-systole (LVIDs), left ventricular internal diameter at end-diastole (LVIDd), left ventricular end-diastolic volume (LVEDV), and end-systolic volume (LVESV). After taking the measurements, the following calculations were made:

(1) $$\begin{array}{c} \text{Left ventricle ejection fraction }\left(\text{LVEF}\right)=\frac{\text{LVEDV}-\text{LDESV}}{\text{LVEDV}}\times 100\%, \end{array}$$

(2) $$\begin{array}{c} \text{LV fractional shortening }\left(\text{FS}\right)=\frac{\text{LVEDD}-\text{LVESD}}{\text{LVEDD}}. \end{array}$$

### 2.4. Morphology and Immunohistochemistry

The removed hearts were immersed in 10% KCl, leaving the hearts arrested during diastole. Hearts were fixed with paraformaldehyde (4%), embedded in paraffin, and sectioned serially (5-6 *μ*m). The slides were stained with hematoxylin-eosin (HE) and wheat germ agglutinin (WGA, Invitrogen, Carlsbad, CA, USA) to observe the morphology of cardiomyocytes. Picric Sirius red (PSR) staining and Masson trichrome staining were performed to detect collagen deposition and fibrosis. The cross-sectional area of myocytes was calculated using Image-Pro Plus 6.0 (Media Cybernetics, Rockville, MD, United States). Immunohistochemical staining of *α*-SMA (Abcam, Cambridge, UK) was performed to evaluate the transformation of fibroblasts to myofibroblasts. The 3-nitrotyrosine (NT) staining and 4 hydroxynonenal (4-HNE) staining (Abcam, Cambridge, UK) were performed to assess the levels of oxidative stress in the myocardium.

### 2.5. Dihydroethidium Staining

Production of intracellular superoxide in the myocardium was detected using in situ dihydroethidium (DHE, Invitrogen Molecular Probes, Eugene, OR, USA) fluorescence. Hearts from different groups were embedded in an optimal cutting temperature compound (OCT Compound, Sakura Finetek USA, Inc., Torrance, CA, United States) and cut into 5 *μ*m sections. Frozen sections were washed with PBS and incubated with 10 *μ*mol/L DHE working solution for 30 min. The images were captured using a fluorescence microscope (DM2500, Leica).

### 2.6. Western Blot Analysis

Hearts and primary cardiomyocyte lysate homogenates were prepared as previously described [24]. Briefly, proteins of whole heart homogenates or cell lysates from different treatments were separated using SDS-polyacrylamide gel electrophoresis (SDS-PAGE) and transferred onto polyvinylidene difluoride membranes (PVDF membranes, Millipore, Billerica, MA, USA). After blocking with 5% nonfat powder milk for 1 h, the membranes were incubated with primary antibodies overnight at 4°C. The primary and secondary antibodies used in our experiments were listed in the Supplementary materials Table S4. The next day, the membranes were washed with the TBST buffer, then incubated with horseradish peroxidase-conjugated secondary antibodies for 1 h at room temperature. The bands were detected using an ImageQuant LAS 4000 Imager (General Electric Co.), and gray-scale value analysis was performed using the Gel-Pro analyzer.

### 2.7. Real-Time Polymerase Chain Reaction Analysis

Total RNA from the frozen heart tissues or primary cardiomyocytes was extracted using the TRIzol Reagent (Invitrogen Life Technologies, USA). Subsequently, the RNA was reverse transcribed to cDNA using a PrimeScript™ RT Master Mix Kit (Takara). Quantitative real-time PCR was performed using a SYBR Green Fast qPCR mix (Takara) with the ABI 7500 Real-Time PCR System (ABI, Carlsbad, CA, USA). The expression levels of the target genes were normalized to that of GAPDH. The primer sequences used in this procedure are listed in Supplementary Table S1 and S2.

### 2.8. Cell Culture and Treatment

Primary neonatal rat cardiomyocytes (NRCMs) were isolated via enzymatic digestion, as described previously. Briefly, hearts from 1- to 3-day-old SD rat pups were minced into tiny pieces and transferred to a centrifuge tube. Ventricles were digested in PBS supplemented with 0.1% trypsin-EDTA and 1 mg/mL collagenase IV (Life Technologies, Darmstadt, Germany) until the digestive enzyme solution was clear. After differential preplating for 2 hours in the incubator, cardiac fibroblasts were attached to the bottom of the petri dish and discarded. Cardiomyocytes were collected and seeded onto different culture plates. The NRCMs were cultured in DMEM supplemented with 10% fetal bovine serum (FBS, GIBCO) and 100 *μ*M Brdu (Sigma-Aldrich) at 37°C and 5% CO_2_ for 24 h. After that, the culture medium was replaced with Dulbecco's modified Eagle medium (DMEM, Life Technologies) containing 2% FBS and 100 *μ*M Brdu. To induce cardiomyocyte hypertrophy, the NRCMs were treated with phenylephrine (PE, Sigma-Aldrich, 50 *μ*mol/L) for 24 h. The LCZ696 dosage was 20 *μ*M. After different treatments, the cells were collected for further analysis.

### 2.9. Cell Surface Area Analysis

Primary neonatal rat cardiomyocytes were seeded onto a confocal dish (D35-20-1.5P, Cellvis, Mountain View, CA) according to the instructions of the manufacturer. Then, the cells were stained with *α*-actinin immunofluorescence. Cell surface area was calculated using Image-Pro Plus 6.0 (Media Cybernetics, Rockville, MD, United States).

### 2.10. Quantification of ROS Generation

Intracellular levels of ROS were assessed using a fluorescence probe, 2′7′-dichlorofluorescein diacetate (DCFH-DA, Abcam, Cambridge, MA, United States) as per the manufacturer's instructions. Briefly, NRCMs were stimulated with different treatments for a specific period. After that, the cells were washed with wash buffer and incubated with 25 *μ*M DCFDA at 37°C in the dark for 45 min, then washed again with wash buffer. Mitochondrial ROS deposition was evaluated using MitoSOX red staining (Invitrogen, Carlsbad, CA, USA). The pictures were captured using a fluorescence microscope (DM3000, Leica).

### 2.11. Cell Apoptosis Assay

Myocardial apoptosis was detected through terminal deoxynucleotidyl transferase-mediated dUTP nick-end labeling (TUNEL) staining using an In Situ Cell Death Detection Kit (Roche Applied Science, Mannheim, Germany) following the manufacturer's instructions. In brief, sections or primary NRCMs were fixed in 4% paraformaldehyde and washed in PBS. After they were permeabilized with 0.3% Triton X-100 and 0.1% sodium citrate, the cells were incubated with the TUNEL reaction mixture for 1 h at 37°C. Then, slides or coverslips were mounted with DAPI (Invitrogen, Carlsbad, CA, USA). Pictures were taken using a fluorescence microscope (Leica DM3000, Germany).

### 2.12. Transfection

Small interference RNA (siRNA) for Sirt3 (si-Sirt3) and negative control siRNA (si-NC) were purchased from RiboBio Co. Ltd. (Shanghai, China). The interference sequences are listed in the Supplementary Table S3. NRCMs were transfected with the siRNAs using Lipofectamine RNAiMAX (Invitrogen) according to the manufacturer's instructions. Knockdown efficiency was assessed using Western blot assay (Supplementary Figure S1). Cells transfected with Sirt3 siRNA were then treated with or without PE (50 *μ*M) for 24 h.

### 2.13. Statistical Analysis

Data analysis was performed using GraphPad Prism 8.0 (GraphPad Software, San Diego, CA, United States). All values are presented as mean ± standard error of the mean (SEM). All the data acquired from our experiments are in accordance with normal distribution. Differences between two groups were analyzed using unpaired Student's *t*-test. One-way ANOVA followed by Bonferroni post hoc test was used for multiple-group comparisons. *p* value < 0.05 was considered statistically significant.

## 3. Results

### 3.1. LCZ696 Improves Cardiac Function and Alleviates Cardiac Hypertrophy in Pressure Overload-Induced Cardiac Remodeling

**Figure 1:**
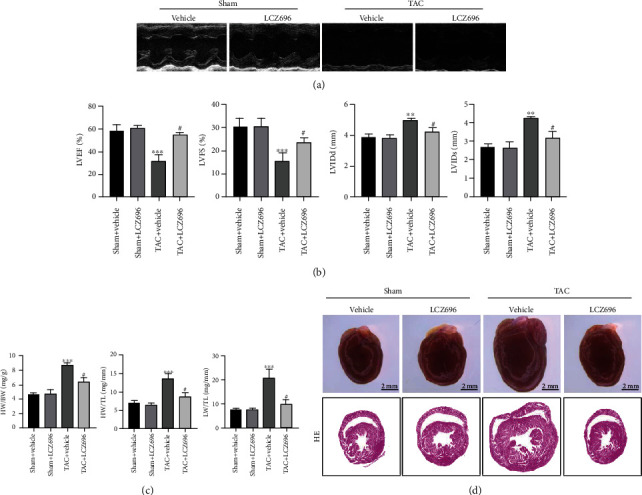
LCZ696 improved cardiac function in pressure overload-induced cardiac hypertrophy. (a) Representative images of echocardiography for sham or TAC mice treated with vehicle or LCZ696 for 4 weeks. (b) Cardiac function as determined by echocardiography (*n* = 6 mice per group). (c) Statistics analysis of HW/BW, HW/TL, and LW/TL for different groups (*n* = 6 mice per group). (d) Anatomical view of the whole heart in different groups under a dissecting microscope and representative images of cross-sectional HE staining. Data are presented as mean ± SEM. ^∗∗∗^*p* < 0.001 vs. sham+vehicle group, ^#^*p* < 0.05 vs. TAC+vehicle group. BW: body weight; HW: heart weight; LW: lung weight; TL: tibia length; HE: hematoxylin-eosin; LVEF: left ventricular ejection fraction; LVFS: left ventricular fractional shortening; LVIDd: left ventricular end-diastolic diameter; LVIDs: left ventricular end-systolic diameter; TAC: transverse aortic constriction.

**Figure 2:**
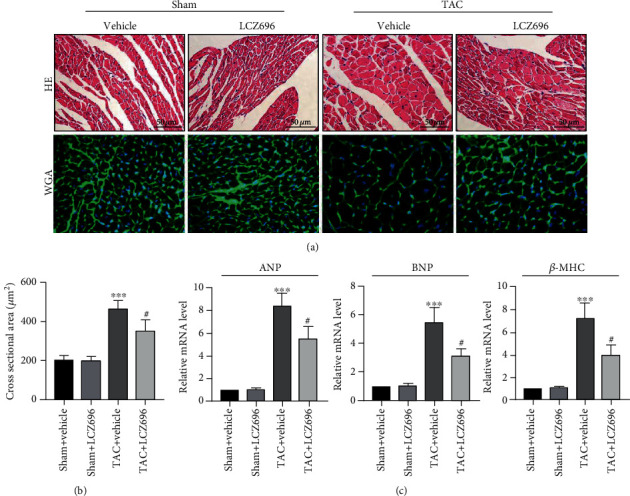
LCZ696 treatment mitigated TAC-induced pathological cardiac hypertrophy. (a) Representative cross-sectional HE staining images and WGA staining images for the sham or TAC mouse hearts treated with vehicle or LCZ696 for 4 weeks. (b) Statistics analysis of cross-sectional areas of cardiomyocytes based on HE staining (*n* > 100 cells per group). (c) mRNA levels of ANP and BNP in different treatment groups. mRNA levels are normalized to the GAPDH mRNA level and converted to fold change relative to the sham+vehicle group (*n* = 6 mice per group). Data are presented as mean ± SEM. ^∗∗∗^*p* < 0.001 vs. the sham+vehicle group, ^#^*p* < 0.05 vs. the TAC+vehicle group. ANP: atrial natriuretic peptide; BNP: brain natriuretic peptide; WGA: wheat germ agglutinin; *β*-MHC: myosin heavy chain *β*.

To determine the effect of LCZ696 on pathological cardiac hypertrophy, wild type mice were administered with LCZ696 via oral gavage at a dose of 20 mg/kg/d for 4 weeks. After 4 weeks of aortic constriction, the mice were anaesthetized, and echocardiography was performed. Administration of LCZ696 increased ejection fraction and fractional shortening (Figures 1(a) and 1(b)), which were both dramatically decreased following TAC surgery. Similarly, TAC caused marked pathological cardiac hypertrophy as indicated by increased ratios of heart weight (HW) to body weight (BW), lung weight (LW) to BW, and HW to tibia length (TL). LCZ696 treatment reduced these ratios, suggesting improved cardiac hypertrophy contractile dysfunction. General view under the microscope and HE staining of the cardiac cross-section also confirmed that TAC-induced cardiac dilation was significantly inhibited by LCZ696 (Figure 1(d)). Furthermore, HE and WGA staining showed that LCZ696 substantially reduced the cross-sectional area of cardiomyocytes (Figures 2(a) and 2(b)). Also, the mRNA markers of cardiac hypertrophy, including atrial natriuretic peptide (ANP), brain natriuretic peptide (BNP), and *β*-myosin heavy chain (*β*-MHC), also referred to as fetal genes, were overtly surged in TAC-primed hearts compared with the sham group. Expectedly, LCZ696 treatment significantly decreased the expression levels of these fetal genes (Figure 2(c)).

### 3.2. LCZ696 Attenuates TAC-Induced Cardiac Fibrosis

**Figure 3:**
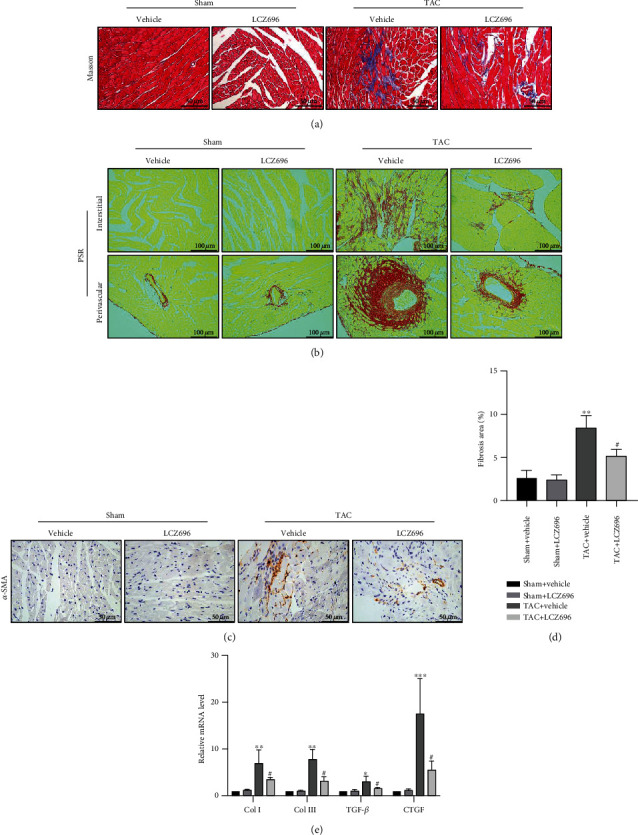
LCZ696 treatment attenuated TAC-induced cardiac fibrosis. (a) Representative images of Masson trichrome staining in different treatment groups. (b) Representative images of Picric Sirius red (PSR) staining of cardiac interstitial and perivascular regions in different treatment groups. (c) Representative immunohistochemical staining of *α*-SMA for myofibroblasts. (d) Quantification of the percentage of left ventricular fibrosis area from (b) (*n* = 6 mice per group). (e) mRNA levels of collagen I, collagen III, TGF-*β*, and CTGF in the indicated groups (*n* = 6 mice per group). The results are normalized against GAPDH and converted to fold change relative to the sham+vehicle group (*n* = 6 mice per group). Data are presented as mean ± SEM. ^∗∗∗^*p* < 0.001 vs. the sham+vehicle group, ^#^*p* < 0.05 vs. the TAC+vehicle group.

Myocardial fibrosis is a crucial feature in pathological cardiac remodeling, which results in impaired cardiac compliance and a decline in the pumping function of the heart. These together promote the progression of hypertrophy to heart failure. Herein, we estimated the effect of LCZ696 in TAC-induced cardiac fibrosis, a classic feature of pathological cardiac hypertrophy, to further elucidate the role of LCZ696 in maladaptive cardiac hypertrophy. Picric Sirius red (PSR) staining and Masson's trichrome staining showed that no significant collagen deposition occurred in the sham group. However, the hearts of TAC mice exhibited severe collagen deposition both in interstitial and in perivascular areas (Figures 3(a) and 3(b)). As expected, LZC696 treatment significantly attenuated TAC-induced cardiac fibrosis (Figure 3(d)). Furthermore, immunohistochemistry staining revealed that LCZ696 treatment markedly alleviated *α*-SMA deposition, which is the hallmark of the transformation of fibroblasts to myofibroblasts. Moreover, we detected the transcription levels of fibrotic markers, including collagen I, collagen III, TGF-*β*, and CTGF. TAC-induced upregulation of these genes was overtly reversed by LCZ696 treatment (Figure 3(e)).

### 3.3. LCZ696 Mitigates TAC-Induced Myocardium Oxidative Stress and Cellular Apoptosis

**Figure 4:**
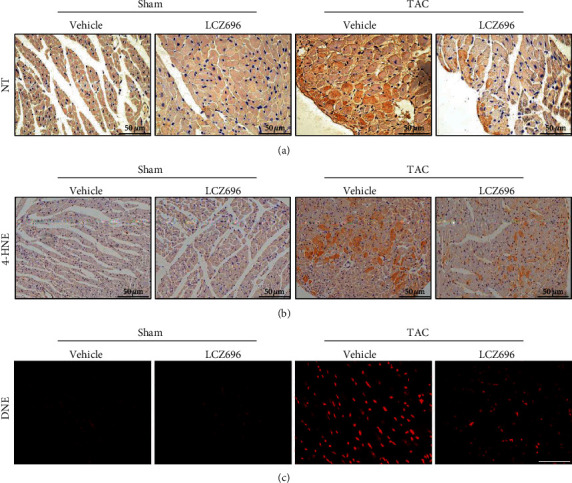
LCZ696 treatment mitigated TAC-induced oxidative stress. (a) Representative immunohistochemical staining images of nitrotyrosine (NT) performed to assess myocardial nitrotyrosine production in different groups. (b) Representative immunohistochemical staining images of 4 hydroxynonenal (4-HNE) performed to assess myocardial lipid peroxidation. (c) Representative immunofluorescence staining images of dihydroethidium (DHE) performed to assess myocardial ROS accumulation.

**Figure 5:**
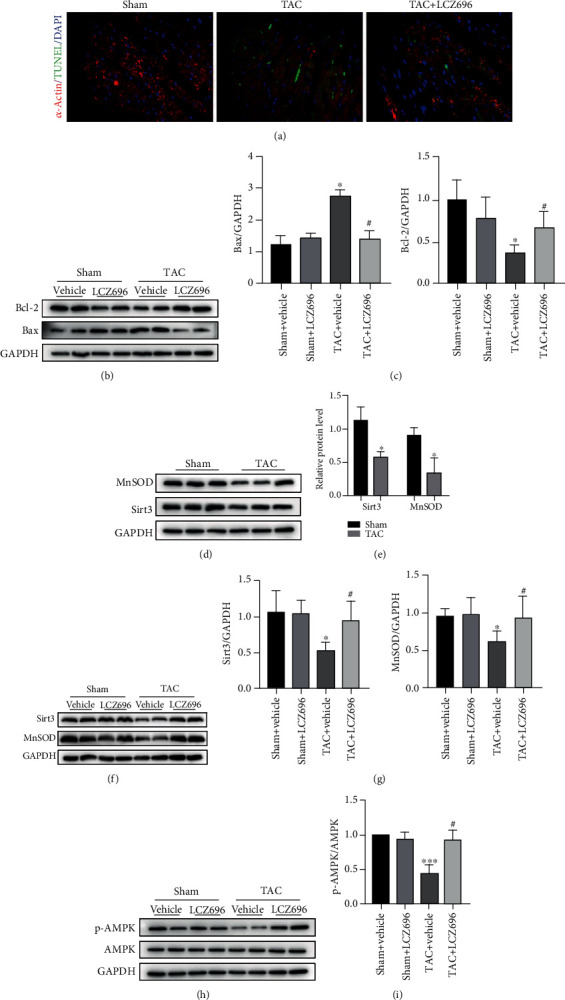
LCZ696 treatment alleviated TAC-induced myocardial apoptosis. (a) Representative TUNEL staining images performed to assess myocardial apoptosis in mouse hearts in different groups. (b, c) Western blot analysis and quantitative analysis of Bcl-2 and Bax protein levels in different cardiac homogenates. (d) The protein expression of Sirt3 and MnSOD levels as detected by Western blot. (e) Histograms showing the quantitative expression of Sirt3 and MnSOD, *n* = 3. (f) Expression levels of Sirt3 and MnSOD in hearts from different groups. (g) Histogram of the quantitative analysis of the data in (f), after normalization to the expression of GAPDH, *n* = 3. (h) Western blot analysis of p-AMPK and AMPK protein levels in different cardiac homogenates. (i) Quantitative analysis of the ratio of p-AMPK/AMPK, *n* = 3. ^∗^*p* < 0.05 vs. the sham+vehicle group, ^∗∗∗^*p* < 0.001 vs. the sham+vehicle group, ^#^*p* < 0.05 vs. the TAC+vehicle group.

Given that oxidative stress can deteriorate cardiac hypertrophy and heart failure [11–13], we examined the effect of LCZ696 on TAC-induced hearts for oxidative stress damage. Pathological hypertrophic stimuli remarkably triggered the production of peroxide byproducts, such as nitrotyrosine (NT) and 4 hydroxynonenal (4-HNE) and superoxide accumulation, as indicated by DHE staining (Figures 4(a)–4(c)). Consistent with our speculations, LCZ696 reduced the deposition of ROS in the myocardium as a result of pressure overload. Furthermore, we assessed myocardial apoptosis using terminal deoxynucleotidyl transferase-mediated dUTP nick-end labeling (TUNEL) assay. LCZ696 attenuated cardiomyocyte apoptosis, as revealed by TUNEL staining (Figure 5(a)). In addition, we examined Bax and Bcl-2 protein levels in different groups, which are both protein markers for apoptosis detection. As expected, LCZ696 remarkably decreased the level of Bax and Bcl-2 expression induced by TAC (Figures 5(b) and 5(c)).

Given that LCZ696 can exert antihypertrophic and antifibrosis effects and attenuate oxidative stress, we further explored the antioxidant reagents in the TAC-induced mice. Pathological hypertrophic stimuli overtly reduced the expression level of MnSOD and Sirt3, whereas LCZ696 treatment distinctly reversed the downregulation of MnSOD and Sirt3 (Figures 5(d)–5(g)). Moreover, we found that LCZ696 upregulated the phosphorylation of AMPK, which was inhibited by pressure overload (Figures 5(h) and 5(i)). Given this, we hypothesized that LCZ696 might exert an antihypertrophic effect by modulating Sirt3 in an AMPK-dependent manner. Thus, we conducted *in vitro* experiments to confirm our speculations.

### 3.4. LCZ696 Blocks the Hypertrophic Response and Alleviates Oxidative Stress and Apoptosis in NRCMs Stimulated with Phenylephrine

**Figure 6:**
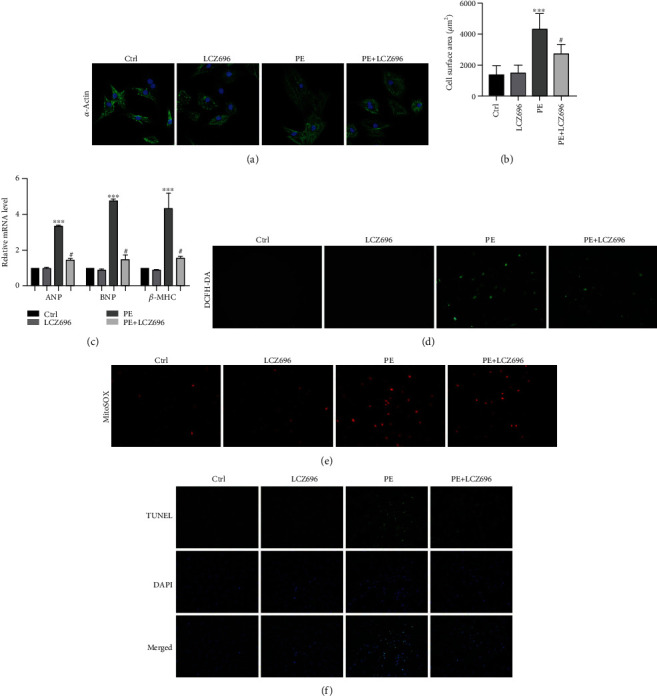
LCZ696 blocked the hypertrophic response in NRCMs. (a) Immunofluorescence staining of sarcomeric alpha-actinin (*α*-actinin) in primary neonatal rat cardiomyocytes (NRCMs) in the indicated treatment groups. (b) The cell surface area of the indicated groups (*n* > 50 cells per experimental group). (c) mRNA levels of *ANP*, *BNP*, and *β-MHC* in different treatment groups. The results were normalized against *GAPDH* and converted to fold change relative to the ctrl group. (d) Intracellular ROS levels in primary cardiomyocytes were quantified with DCFH-DA staining. (e) Mitochondrial ROS levels in primary cardiomyocytes were measured by MitoSOX staining. (f) Representative TUNEL staining of primary cardiomyocytes in different treatment groups. Data are presented as mean ± SEM. ^∗∗∗^*p* < 0.001 vs. the control group, ^#^*p* < 0.05 vs. the PE treatment group.

Here, we verified the effect of LCZ696 on NRCMs. Firstly, we conducted a cell viability assay to evaluate effect of different concentrations of LCZ696 on cardiomyocyte viability (Supplementary Figure S2), and we used 20 *μ*M LCZ696 to incubate with NRCMs in the following *in vitro* experiments. Then, phenylephrine (PE, 50 *μ*mol/L) was used to stimulate NRCMs for 24 h to imitate the process of cardiac hypertrophy *in vitro*. As expected, LCZ696 reduced the surface area of hypertrophied cardiomyocytes, as demonstrated by *α*-actinin staining (Figures 6(a) and 6(b)). Likewise, transcription levels of other hypertrophic markers, such as atrial natriuretic peptide (ANP), brain natriuretic peptide (BNP), and *β*-myosin heavy chain (*β*-MHC), were significantly increased in the PE-treated cardiomyocytes compared with the control group. LCZ696 overtly reduced the expression of these fetal genes (Figure 6(c)).

We also monitored the oxidative damage induced by phenylephrine in NRCMs. LCZ696 reduced the intensity of DCFH fluorescence in NRCMs following PE treatment. Furthermore, we detected the levels of superoxide in the mitochondria via MitoSOX staining (Life Technologies, Darmstadt, Germany). As anticipated, LCZ696 significantly reduced the oxidative stress in the mitochondria (Figure 6(e)). Besides, LCZ696 reduced cardiomyocyte apoptosis, as witnessed by TUNEL staining (Figure 6(f)).

### 3.5. LCZ696 Represses Cardiac Hypertrophy by Upregulating Sirt3

**Figure 7:**
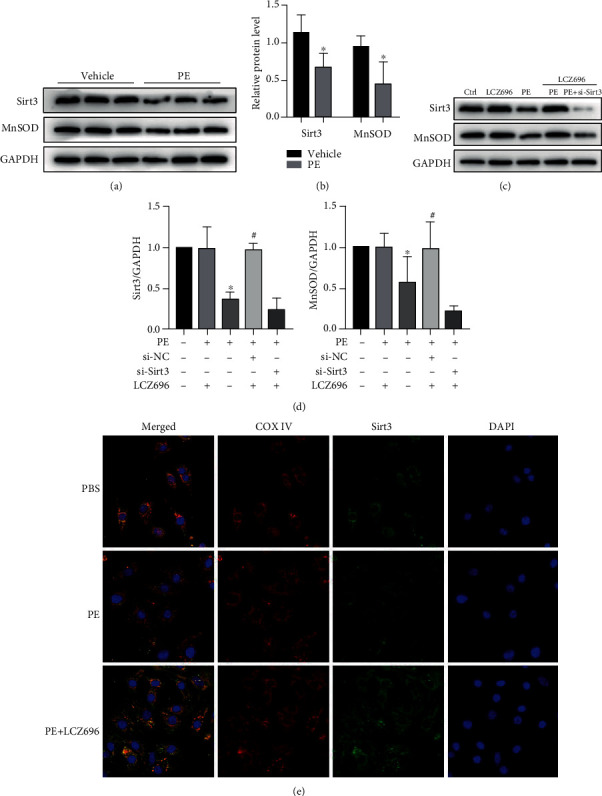
LCZ696 upregulated Sirt3 expression. (a) NRCMs were treated with 50 *μ*M PE for 24 h, and the expression of Sirt3 and MnSOD were detected by Western blotting. (b) Histograms showing the quantitative analysis of Sirt3 and MnSOD, *n* = 3. (c) NRCMS were transfected with small interfering RNA targeting Sirt3 (si-Sirt3, 50 nM) of scramble small interfering RNA (si-NC,50 nM) for 48 h and then treated as indicated. The expression of Sirt3 and MnSOD was determined with immunoblotting. (d) Histogram of the quantitative analysis of the data in (c), after normalization to the GAPDH levels, *n* = 3. (e) Representative images of immunofluorescence staining for Sirt3 and mitochondrial marker (COX IV) in NRCMs. ^∗^*p* < 0.05 vs. the control group, ^#^*p* < 0.05 vs. the PE treatment group.

Here, we explored the underlying mechanisms of the antihypertrophic effect of LCZ696 in a PE-induced cardiomyocyte hypertrophy model. First, we tested the expression level of Sirt3 and MnSOD. Consistent with the abovementioned results (Figure 5), hypertrophic stimuli inhibited Sirt3 and MnSOD expressions following PE treatment. With that, we introduced small interfering RNA (siRNA) to knock down the Sirt3 expression. LCZ696 did not upregulate the expression of MnSOD after the Sirt3 knockdown (Figure 7(c)). We then determined the cellular localization of Sirt3 in cardiomyocytes. Sirt3 was colocalized with mitochondrial marker COX IV and decreased in response to PE stimulation (Figure 7(e)). LCZ696 upregulated Sirt3 expression following PE treatment. Notably, LCZ696 did not upregulate the level of Sirt3 basal expression.

### 3.6. Sirt3 Knockdown Abolishes the Protective Effect of LCZ696 on Cardiomyocyte Hypertrophy

**Figure 8:**
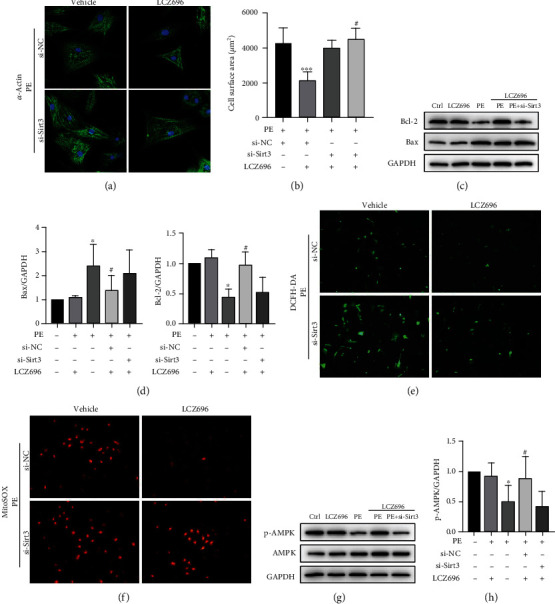
Sirt3 knockdown abolished the antihypertrophic effect of LCZ696 in PE-induced cardiomyocyte hypertrophy. (a) Immunofluorescence staining of *α*-actinin in primary neonatal rat cardiomyocytes (NRCMs) in the indicated groups. (b) The cell surface area in the indicated groups (*n* > 50 cells per experimental group), ^∗∗∗^*p* < 0.001 vs. the si-NC+vehicle group, ^#^*p* < 0.05 vs. the si-NC+LCZ696 treatment group. (c, d) Western blot analysis and quantitative analysis of Bcl-2 and Bax protein levels from different treatment groups. (e) Representative images of DCFH-DA staining in NRCMs. (f) Representative images of MitoSOX staining in NRCMs. (g, h) Western blot analysis of p-AMPK and AMPK protein levels in different treatment NRCMs and quantitative analysis of the ratio of p-AMPK/AMPK, *n* = 3. ^∗^*p* < 0.05 vs. control group, ^#^*p* < 0.05 vs. PE treatment group.

Sirt3 deficiency impaired the cardioprotective effect of LCZ696 in alleviating hypertrophic response of cardiomyocytes following PE treatment (Figure 8(a)). In addition, LCZ696 did not influence the expression of Bax and Bcl-2 under Sirt3 deficiency conditions (Figures 8(c) and 8(d)). Furthermore, Sirt3 knockdown abolished the alleviation of oxidative stress mediated by LCZ696 (Figure 8(e)). To verify the involvement of Sirt3 in the LCZ696-induced antioxidant property, we performed MitoSOX staining to examine the generation of superoxide by the mitochondria. Sirt3 deficiency aggravated PE-induced oxidative stress and abrogated the protective effect of LCZ696 in mitochondrial ROS generation. Phenylephrine (PE) stimulation decreased the ratio of p-AMPK/AMPK, which was reversed by LCZ696. However, Sirt3 knockdown impeded this process, indicating that the cardioprotective effect of LCZ696 was partly involved in the upregulation of phospho-AMPK.

## 4. Discussion

In recent decades, heart failure-associated deaths have increased drastically, prompting researchers to focus on conducting studies that can solve the problem. Nevertheless, heart failure is the last battlefield in cardiovascular therapy and has always been challenging to tackle [25, 26]. Many pathological conditions, such as acute myocardial infarction, chronic uncontrolled hypertension, and diabetes, can contribute to the occurrence of heart failure. Among them, cardiac hypertrophy, which is usually caused by pressure or volume overload, is the most common cause of heart failure [1, 2, 27].

LCZ696, also referred to as sacubitril/valsartan, is currently the standard treatment option against the progression of heart failure with reduced ejection fraction [4–7]. Numerous studies have examined the cardioprotective effects of LCZ696 on left ventricular remodeling after myocardial infarction. LCZ696 improves cardiac function by attenuating cardiac fibrosis and MMP-9 expression after ischemia-reperfusion (IR) injury [22, 28, 29]. Moreover, LCZ696 can attenuate cardiac remodeling and cardiac inflammation by alleviating dynamin-related protein 1 (Drp-1) expression in a doxorubicin-induced cardiomyopathy animal model [30]. Although there are several reports on the basic mechanisms underlying the effectiveness of LCZ696, candidate mechanisms responsible for the direct protective effect of LCZ696 against cardiomyocyte hypertrophy remain unexplored.

Several studies have confirmed that reactive oxygen species (ROS) can cause adverse effects by oxidizing proteins, lipids, and nucleic acids. Cardiac hypertrophy and oxidative stress are mutually causal, forming a vicious circle. Therefore, breaking the vicious circle could be a vital breakthrough in the treatment of heart failure. However, antioxidant therapy has not been developed. Recent studies have confirmed that impairment of catalase, a major antioxidant enzyme, can aggravate diabetes-induced cardiac toxicity. On the contrary, cardiac-specific overexpression of catalase alleviated aging-related cardiac dysfunction and cardiac hypertrophy [31, 32]. Superoxide dismutases (SOD) are the major antioxidant enzymes responsible for the scavenger of superoxide in the mitochondria. Among them, manganese SOD (MnSOD, SOD2) in the mitochondria plays a pivotal role in redox homeostasis [33]. Manganese superoxide dismutase (MnSOD) is a major superoxide-scavenging enzyme, converting superoxide to hydrogen peroxide, which is in turn hydrolyzed to water by catalase [34].

Sirt3 belongs to CLASS III histone deacetylases (HDACs) and localizes to the mitochondrial matrix, where it may function as a primary stress-responsive protein deacetylase [35]. Many studies have focused on examining the cardioprotective role of Sirt3 in cardiac hypertrophy and heart failure [36]. For example, Sirt3 knockout mice exhibited severe cardiac hypertrophy and fibrosis induced by TAC, and nevertheless, cardiomyocyte-specific overexpression of Sirt3 resisted cardiac fibrosis and oxidative damage following angiotensin II infusion [20]. A study also reported that Sirt3-deficient mice developed dilated cardiomyopathy, and Sirt3 knockout mice exacerbated angiotensin II-induced cardiac hypertrophy [19]. SIRT3 deficiency can aggravate diabetic cardiomyopathy by inactivating Foxo3A-mediated mitophagy and accelerate hypertensive cardiac remodeling by impairing angiogenesis [35, 37]. Besides, Sirt3 silencing can abolish the protective effects of NaHS to reverse Ang II-induced cardiomyocyte hypertrophy and mitochondrial dysfunction as a result of the decline in FOXO3a and SOD2 expression [38, 39]. Studies have shown that many small molecules with Sirt3-activating property can protect against cardiac hypertrophy and heart failure. Honokiol increases Sirt3 activity, thereby alleviating the severity of cardiac hypertrophy [40]. It also reduces oxidative stress induced by melatonin and improves cardiac function by interacting with the Sirt3-dependent pathway [41, 42]. Resveratrol, known as a potent activator of SIRT1, also ameliorates cardiac fibrosis by slightly activating Sirt3 [43]. Sirt3 plays an anticarcinogenic role and functions as a tumor suppressor protein [44]. Clinical studies have shown that the Sirt3 level decreases by 40% in 65-year-old people [45], which may be closely related to the occurrence of many multiple senile diseases, such as hypertension, atherosclerosis, and heart failure.

Given that Sirt3 can play such a crucial role in pathological cardiac remodeling by deacetylating MnSOD [20, 44, 46], we hypothesized that LCZ696 might regulate the expression of Sirt3, mitigate oxidative stress, and thus improve cardiac hypertrophy and heart failure. To verify our hypothesis, we conducted an *in vivo* experiment with transverse aortic constriction, as described earlier. Laparoscopic Transabdominal Cerclage (TAC) mice exhibited deteriorated cardiac dysfunction as evidenced by echocardiography and the ratios of HW/BW, HW/TL, and LW/TL (Figure 1). Moreover, TAC markedly promoted the fibrosis deposition and transdifferentiation from fibroblast to myofibroblast, and as a result, cardiac distensibility declined. Fortunately, LZC696 alleviated myocardium hypertrophy and fibrosis, thereby improving cardiac function. With that, we investigated the mechanisms underlying the role of LCZ696 in cardiac hypertrophy. Pressure overload repressed the expression level of Sirt3 and MnSOD, whereas LCZ696 rescued the decline of Sirt3 and MnSOD. Also, we found that LCZ696 upregulated the phosphorylation of AMPK, whose deficiency could aggravate pressure overload-induced cardiac hypertrophy. Therefore, we hypothesized that LCZ696 might exert an antihypertrophic effect by modulating Sirt3 in an AMPK-dependent manner. To verify this hypothesis, we conducted *in vitro* experiments. Phenylephrine (PE) was used to stimulate neonatal rat cardiomyocytes (NRCMs) to imitate an *in vitro* cardiac hypertrophy model. As explained earlier, LCZ696 blocked the hypertrophic response and alleviated oxidative stress and apoptosis in NRCMs following PE stimulation. In addition, LCZ696 upregulated Sirt3 and MnSOD expression, as well as the ratio of p-AMPK/AMPK. However, Sirt3 silencing abolished the ability of LCZ696 to reverse the PE-induced cardiomyocyte hypertrophy, mitochondrial oxidative stress, and apoptosis, along with the decline in MnSOD expression. Also, Sirt3 deficiency hampered the capacity of LCZ696 to activate the phosphorylation of AMPK. Collectively, these results demonstrate that Sirt3 could be an endogenous negative regulator of cardiac hypertrophy, which protects hearts by suppressing cellular levels of ROS. In addition, LCZ696 might exert antihypertrophic effect by ameliorating oxidative stress via the Sirt3/MnSOD pathway.

## 5. Conclusion

Our results show that Sirt3 can act as a therapeutic target in the treatment of heart failure. Also, the application of specific small molecules for activating Sirt3 can be a novel strategy to hinder the progression of pathological hypertrophy into heart failure. These findings provide new insights into the molecular mechanism of LCZ696 and its novel therapeutic role in the treatment of pathological cardiac remodeling and heart failure.

## Data Availability

The data used to support the findings of this study are available from the corresponding author upon request.

## Supplementary Materials

Supplementary Materials Supplementary Table S1: mouse primer sequences for qRT-PCR. Supplementary Table S2: rat primer sequences for qRT-PCR. Supplementary Table S3: sequences of siRNAs for Sirt3. Supplementary Table S4: primary and secondary antibodies used in the Western blot. Supplementary Figure S1: the effect of different small interference sequences for Sirt3 knockdown. As shown in the figure, sequence 1 (Si-1) and sequence 3 (Si-3) effectively knockdown Sirt3 expression and Si-3 works best. As a result, we used Si-3 in our following experiments. Supplementary Figure S2: cell viability assay of different concentrations of LCZ696 in primary cardiomyocytes. Neonatal rat cardiomyocytes were seeded onto a 96-well plate overnight, and different concentrations of LCZ696 (0, 20, 30, 40 *μ*M) were added into culture medium for 24 h. Then, cell viability was measured using a commercial Cell Counting Kit-8 (CCK-8, MedChemExpress, Monmouth Junction, NJ, USA). The concentration of LCZ696 used in our *in vitro* experiment is 20 *μ*M.
