# Supplementary material for: LCZ696 Ameliorates Oxidative Stress and Pressure Overload-Induced Pathological Cardiac Remodeling by Regulating the Sirt3/MnSOD Pathway
Source: Oxid Med Cell Longev. 2020 Sep 17;2020:9815039. doi: 10.1155/2020/9815039 (PMC7519988; doi:10.1155/2020/9815039)
Supplement: Supplementary Materials — Supplementary Table S1: mouse primer sequences for qRT-PCR. Supplementary Table S2: rat primer sequences for qRT-PCR. Supplementary Table S3: sequences of siRNAs for Sirt3. Supplementary Table S4: primary and secondary antibodies used in the Western blot. Supplementary Figure S1: the effect of different small interference sequences for Sirt3 knockdown. As shown in the figure, sequence 1 (Si-1) and sequence 3 (Si-3) effectively knockdown Sirt3 expression and Si-3 works best. As a result, we used Si-3 in our following experiments. Supplementary Figure S2: cell viability assay of different concentrations of LCZ696 in primary cardiomyocytes. Neonatal rat cardiomyocytes were seeded onto a 96-well plate overnight, and different concentrations of LCZ696 (0, 20, 30, 40 μM) were added into culture medium for 24 h. Then, cell viability was measured using a commercial Cell Counting Kit-8 (CCK-8, MedChemExpress, Monmouth Junction, NJ, USA). The concentration of LCZ696 used in our in vitro experiment is 20 μM. [file 9815039.f1.docx]

**LCZ696 Ameliorates Oxidative Stress and Pressure Overload-Induced Pathological Cardiac Remodeling via Regulating Sirt3/MnSOD Pathway**

Shi Peng ^a,1^, Xiao-feng Lu ^a,1^, Yi-ding Qi ^b,1^, Jing Li ^C^, Juan Xu ^a^, Tian-you Yuan ^a^, Xiao-yu Wu ^a^, Yu Ding ^a^, Wen-hua Li ^d^, Gen-qing Zhou ^a^, Yong Wei ^a^ , Jun Li ^a^, Song-wen Chen ^a,*^, Shao-wen Liu ^a,*^

**Supplementary Data**

**Supplementary Table S1.** Mouse primers sequences for qRT-PCR

| Primer | Sequences |
| --- | --- |
| Sirt3 | Forward:5'-TCTATACACAGAACATCGACGG-3'  Reverse:5' -GCATGTAGCTGTTACAAAGGTC-3' |
| ANP | Forward:5'- ACCTGCTAGACCACCTGGAG -3'  Reverse:5'- CCTTGGCTGTTATCTTCGGTACCGG -3' |
| BNP | Forward:5'- GAGGTCACTCCTATCCTCTGG -3'  Reverse:5'- GCCATTTCCTCCGACTTTTCTC -3' |
| β-MHC | Forward:5'- ACTGTCAACACTAAGAGGGTCA -3'  Reverse:5'- TTGGATGATTTGATCTTCCAGGG -3' |
| Collagen I | Forward:5'- ACGAGGTGACAAAGGTGAAACTGG -3'  Reverse:5'- AGAACCTGGAGGACCTGGATTGC -3' |
| Collagen III | Forward:5' CAGGCCAGTGGCAATGTAAAGA 3'  Reverse:5' CTCATTGCCTTGCGTGTTTGATA 3' |
| TGF-β | Forward:5' AACACCCAGCCCATTTAC 3'  Reverse:5' GGTTGGCGTTCTCATCTAC 3' |
| CTGF | Forward:5' ACCCGAGTTACCAATGACAATACC 3'  Reverse:5' CCGCAGAACTTAGCCCTGTATG 3' |
| GAPDH | Forward:5'-AGGAGTAAGAAACCCTGGAC-3'  Reverse:5'-CTGGGATGGAATTGTGAG-3' |

**Supplementary Table S2.** Rat primers sequences for qRT-PCR

| Primer | Sequences |
| --- | --- |
| Sirt3 | Forward:5'-TCAGCAGTATGACATCCCGTACCC-3'  Reverse:5' -CGTGAAGCAGCCGAAGGAAGTAG-3' |
| ANP | Forward:5'- GAGCGAGCAGACCGATGAAGC-3'  Reverse:5'- TCCATCTCTCTGAGACGGGTTGAC -3' |
| BNP | Forward:5'- AGTCTCCAGAACAATCCACGATGC -3'  Reverse:5'- GCCTTGGTCCTTTGAGAGCTGTC -3' |
| β-MHC | Forward:5'- CCAGAACACCAGCCTCATCAACC -3'  Reverse:5'- CACCGCCTCCTCCACCTCTG -3' |
| GAPDH | Forward:5'-AGGAGTAAGAAACCCTGGAC-3'  Reverse:5'-CTGGGATGGAATTGTGAG-3' |

**Supplementary Table S3.** Sequences of siRNAs for Sirt3

| siRNAs | Sequences |
| --- | --- |
| si001 | GAAAGATGTGGTCCAGCTA |
| si003 | CAGCAAGGTTCTTACTACA |
| si004 | AAAATGGAAAGCTGGATGG |
| negative control | GGCTCTAGAAAAGCCTATGC |

**Supplementary Table S4.** Primary and Secondary antibodies used in the Western Blot

| Target protein | Antibody Supplier Dilution |
| --- | --- |
| GAPDH | Cell Signaling Technology 1:1000 |
| Bax | Cell Signaling Technology 1:1000 |
| Bcl-2 | Abcam 1:1000 |
| Sirt3 | Cell Signaling Technology 1:1000 |
| MnSOD | Cell Signaling Technology 1:1000 |
| Phospho-AMPKα (Thr172) | Cell Signaling Technology 1:1000 |
| AMPKα | Cell Signaling Technology 1:1000 |
| Anti-rabbit IgG, HRP-linked | Cell Signaling Technology 1:5000 |
|  |  |

**Supplementary Figure S1.**


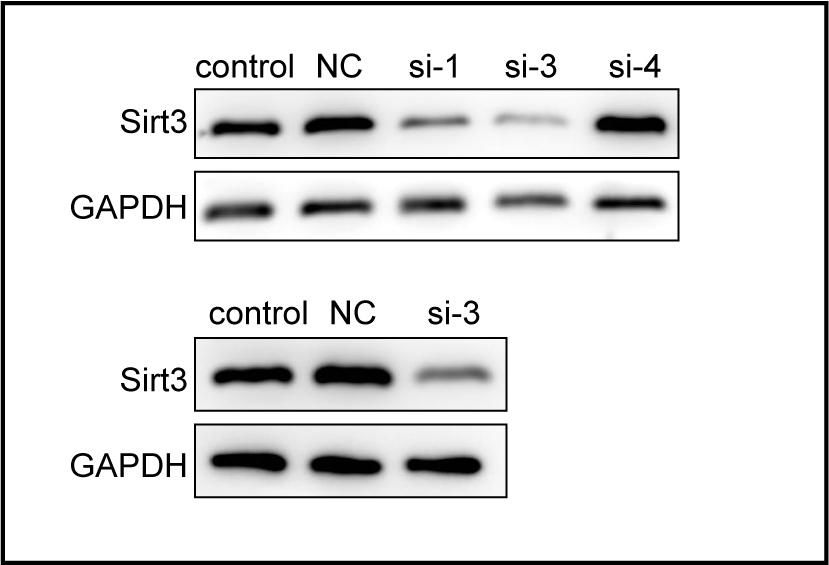


**Supplementary Figure S1. The effect of different small interference sequences for Sirt3 knockdown.** As shown in the figure, sequence1 (Si-1) and suquence3 (Si-3) effectively knockdown Sirt3 expression and Si-3 works best. As a result, we used Si-3 in our following experiments.

**Supplementary Figure S2.**


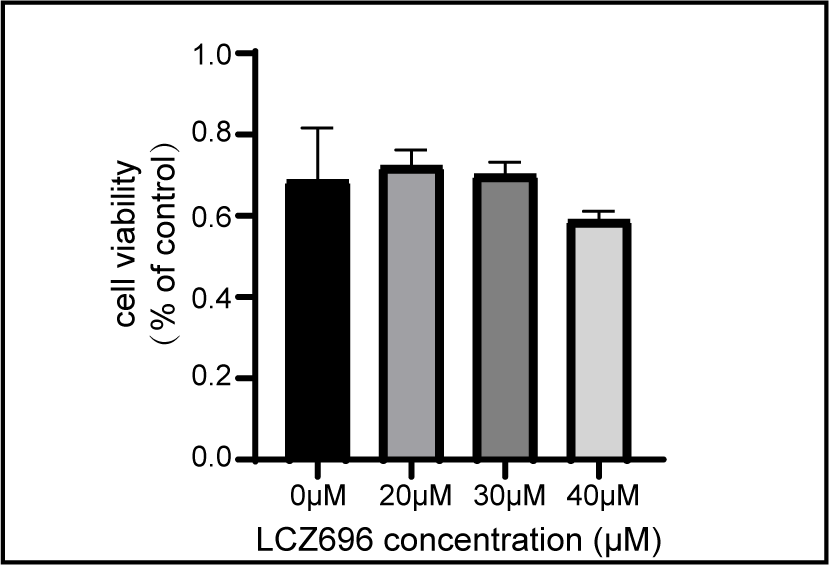


**Supplementary Figure S2. Cell viability assay of different concentrations of LCZ696 in primary cardiomyocytes.** Neonatal rat cardiomyocytes were seeded onto a 96-well plate overnight and different concentrations of LCZ696 (0, 20, 30, 40 μM) were added into culture medium for 24 h. Then, cell viability was measured using a commercial Cell Counting Kit-8 (CCK-8, MedChemExpress , Monmouth Junction, NJ, USA). The concentration of LCZ696 used in our *in vitro* experiment is 20 μM.
